# Patterns of hospital utilization in the Unified Health System in six Brazilian capitals: comparison between the year before and the first six first months of the COVID-19 pandemic

**DOI:** 10.1186/s12913-021-07006-x

**Published:** 2021-09-17

**Authors:** Margareth Crisóstomo Portela, Claudia Cristina de Aguiar Pereira, Sheyla Maria Lemos Lima, Carla Lourenço Tavares de Andrade, Mônica Martins

**Affiliations:** Department of Health Administration and Planning, Sergio Arouca National School of Public Health, Oswaldo Cruz Foundation, Rio de Janeiro, Brazil

**Keywords:** COVID-19, Hospitalizations, Hospital mortality, SUS

## Abstract

**Objective:**

To analyze the temporal evolution of the pattern of hospital use in the context of the COVID-19 pandemic in Brazil.

**Methods:**

This retrospective observational study compared hospital use and mortality in the Brazilian Unified Health System (SUS) in the first six months of the COVID-19 pandemic with the year before the onset of the pandemic in six Brazilian capitals (São Paulo, Rio de Janeiro, Manaus, Fortaleza, Recife, and Brasilia). It was based on secondary administrative data from the SUS Hospital Information System (SIH), focusing on the number of hospitalizations per fortnight, age, and gender of patients, hospital length of stay, and the proportions of surgical, elective, with the use of ICU, and resulting in death hospitalizations. It also compared the number of hospitalizations and mortality related to frequent diagnostic groups.

**Results:**

A significant drop was identified in the number of hospitalizations as of March 2020, with the first peak of COVID-19 hospitalizations in five capitals recorded in May 2020. In the six capitals, we observed significant reductions in the mean number of hospitalizations per fortnight from the beginning of the pandemic. We also identified an increase in the mean age of the patients and the proportion of male patients. The proportion of surgical and elective hospitalizations dropped significantly in all capitals, while the proportion of hospitalizations with ICU use increased significantly. Significant increases in-hospital mortality were also recorded in the six capitals with the pandemic, including or excluding COVID-19 hospitalizations from the comparison.

**Conclusion:**

The pandemic caused changes in the pattern of use and hospital indicators in the first six months in the cities considered, evidencing the need for attention to diseases with a hospital production altered by the COVID-19 course and health system performance problems in the face of challenges.

## Introduction

In general, the pattern of use of hospital services depends on the characteristics of the population’s health needs and the provision of services. While adequate and timely access to other levels of care can avoid unnecessary or excessive use, effective hospital care plays an important role, whether in situations of deteriorating chronic conditions, elderly patients, or emergency cases. Dependent on intensive hospital care, this demand compounded in the context of the COVID-19 pandemic potentially impacts patients’ access to other needs and the effectiveness of the care provided. Considering the perennial challenge about varying practice and utilization pattern [1], the pandemic setting has also been envisioned as a “trial” to sensitize people to the problems arising from overuse and low-value care affecting the quality and sustainability of health systems [2, 3]. However, this same scenario provides other elements that can contribute to insufficient use and inequalities in access and outcomes [3, 4].

Countless studies describe the excessive number of deaths from COVID-19 in the recent period, but also due to other causes [5–8], some even emphasizing a higher number of unassisted home-bound deaths [9, 10]. Woof et al. [5] estimated the excess of 87,001 deaths, of which 65% were attributable to COVID-19, and 35% were unexplained. While data may show some inaccuracies, it has been suggested that people avoid seeking care for fear of infection. On the other hand, social distancing measures may affect the reduction of vehicle traffic and, consequently, morbimortality due to accidents and use of emergency services [11]. Changes in hospital morbidity are expected besides the mortality profile. For example, surgeries represented 50% of hospital capacity in the U.S. between March and April 2020, partly due to the suspension of elective procedures and other non-urgent care [12]. Concomitantly, there is evidence of a drop in acute clinical hospitalizations during pandemic escalation, such as stroke, acute myocardial infarction, or diabetes, raising questions about the impact on health conditions and people’s access with needs unrelated to COVID-19 [12–17].

From the viewpoint of service and care organization, cardiovascular problems, cancer, and elective surgery care plans were seemingly postponed, representing a pent-up demand to be met in the medium term [4, 16, 18–20]. The search for hospital care for acute problems seems to have grown when the outbreak was minimized. However, this has not yet been widely observed [15] in the case of chronic diseases. While part of these hospitalizations can be considered unnecessary due to excessive use [1], the continuity and coordination of care for these patients is a concern. Some authors have even been predicting the deterioration of the health condition and possible loss of care effectiveness [21].

Thereby, considering the challenges for providing hospital care to COVID-19 and non-COVID-19 patients, and notably the unappropriated and uncontrolled management of this sanitary crisis in the Brazilian context, damage has resulted from non-COVID-19 healthcare unmet needs, representing, among other aspects, less access to adequate and timely care, and greater risk of adverse results. In different moments, the COVID-19 pandemic in Brazil led the health system to the exhaustion of its installed capacity. Capturing the extension of the damage and building knowledge to support choices regarding healthcare reorganization to deal with routine, unmet needs, and Long Covid new demands imposed is necessary. Among other elements, it is vital to profile the utilization pattern in the hospital network of the Unified Health System (SUS) during this pandemic. To some extent, it translates into changes in the behavior of indicators for the use of hospital services, especially in the number of hospitalizations, intensive care use, length of stay, case profile, and hospital mortality. Based on this assumption, this paper aims to analyze the temporal evolution of the pattern of hospital use in the SUS, in the preceding setting, and during the COVID-19 pandemic, in Brazilian capitals.

## Methods

This was a retrospective observational study comparing hospital production and mortality in the SUS in the first six months (February 23rd – September 5th, 2020) of the COVID-19 pandemic in Brazil with the year before the onset of the pandemic in six Brazilian capitals. The analysis was based on ordinary administrative secondary data from the SUS Hospital Information System (SIH), obtained from the DATASUS website on January 18, 2021. SIH has national coverage and includes data on hospitalizations in the SUS, including demographic and clinical variables related to the care process, payment amount, and outcome.

According to SIVEP-Gripe, a public and open-access database of Severe Acute Respiratory Illness records (including COVID-19) collected by the Brazilian Ministry of Health, the cities of São Paulo, Rio de Janeiro, Brasília, Fortaleza, and Recife, presented the highest number of Covid-19 hospitalizations in the period focused. Furthermore, we selected Manaus (ranked number 8 in number of hospitalizations) because of the severe problems faced by the city in dealing with the pandemic, including lack of hospital beds, supplies, and medical personnel. All six capitals are included in the group of the ten largest capitals in the country, and together account for approximately 13.7% of the Brazilian population.

The SIH microdata files of each state and Federal District, corresponding to 2019 and for the period from January to November of 2020, were extracted from DATASUS website (http://www2.datasus.gov.br/DATASUS/index.php?area=0901&item=1&acao=25). Accounting for data beyond September 2020, allowed for mitigating the loss of hospitalizations with longer lengths of stay and delays in the flow of information for inclusion in the SIH. From the database aggregating the files, observations related to obstetric hospitalizations were excluded, considering the code of chapter XV of the International Statistical Classification of Diseases and Related Health Problems, 10th Revision (ICD-10) registered in the variable ‘primary diagnosis’. All hospitalizations of children under 18 and older adults aged 100 years, or more were also excluded.

The temporal segmentation considered as cutoff, the date of the first official COVID-19 case record in Brazil (February 26th, 2020), in the ninth epidemiological week of the year, which started on February 23rd. Fortnightly periods were defined starting from the ninth epidemiological week of 2020, in which the COVID-19 hospitalizations began, behind and ahead: in the first case, 26 fortnights beginning on February 24th, 2019, and the last ending on February 22nd, 2020; in the second case, 14 fortnights starting on February 23rd and ending on September 5th, 2020. Chronologically numbered, fortnights 1–26 provided the baseline for comparing pandemic period indicators, starting at fortnight 27.

After data management, the capitals of interest were separated using the variable ‘municipality of movement’ that informs the place where the hospitalization took place, considering their respective codes in the classification of the Brazilian Institute of Geography and Statistics (IBGE).

In more global terms, comparisons between the period of the pandemic and the baseline established by the year preceding its onset were made in each capital, considering the number of hospitalizations per fortnight, patients’ age and gender, hospitalization’s length of stay, the proportion of surgical and elective hospitalizations, the proportion of hospitalizations with the use of intensive care unit (ICU), and the proportion of hospitalizations that resulted in death. For the last two indicators, the comparisons were made considering both all hospitalizations and non-COVID-19 only hospitalizations during the pandemic. Surgical and elective hospitalizations were defined by the ‘specialty’ and ‘admission type’ variables. Following the technical guidelines of the SIH, the occurrence of COVID-19, in turn, was determined in all hospitalizations with the primary diagnosis or one of the secondary diagnoses specified as B34.2 (coronavirus infection of unspecified location) according to ICD-10, besides those whose procedure was “03.03.01.022-3 - NEW CORONAVIRUS COVID 19 INFECTION TREATMENT”, in force as of April 2020 [22].

We then selected the ten primary diagnoses that appeared among the most frequent in SUS hospitalizations in the six capitals, comparing, before and during the first six months of the pandemic, the number of hospitalizations and the observed hospital mortality.

The analyses were descriptive, and we obtained means, standard deviations, and medians of the numerical variables and absolute and relative frequencies of categorized variables. Means were compared with the t-test, and Fisher’s exact test was employed to identify associations between events such as deaths or use of ICU with the moment of hospitalization, before or during the pandemic. The criterion for statistical significance was defined as α = 0.05.

Statistical control charts were also used [23–25] to visualize surgical and elective hospitalizations, the use of ICU, and death during the 40 fortnights, assuming the behavior pattern of the indicators during the first 26 fortnights as a reference for defining the statistical control “zone”. Such charts presuppose statistical control in the trend of indicators over time, which can be broken by positive or negative events. They include the mean value of the indicator and the statistical control “zone” conventionally bounded by three standard deviations below and above the mean value. Changes in the current pattern can be configured in terms of the deviations highlighted in the magnitude or variability of the indicator. The technique is simple and allows visualizing effects on event indicators such as the pandemic.

Managing data, obtaining descriptive statistics, and drawing charts were facilitated by the Statistical Analysis System (SAS®) package. For statistical control charts, ‘PROC SHEWHART’ was used with the command ‘pchart’, used for proportions, considering the binomial distribution.

## Results

Looking at the number of hospitalizations between February 24th, 2019, and September 5th, 2020 in the six capitals (Table 1), a significant drop is observed from the fortnight that begins on March 22nd, 2020, with COVID-19 hospitalizations in May 2020, especially between the days 03 and 16, reaching proportions of 30.9, 26.8, 45.7, 43.6, and 31.9%, respectively, in São Paulo, Rio de Janeiro, Manaus, Fortaleza, and Recife. In Brasília, the first peak of COVID-19 hospitalizations occurred comparatively later, between the end of July and early August, corresponding to 24.5% of the hospitalizations.

**Table 1:** Total hospitalizations and hospitalizations due to COVID-19 in six Brazilian capitals in the previous year and during the first six months of the pandemic, 24/02/2019 to 05/09/2020

Fortnight			São Paulo			Rio de Janeiro			Manaus			Fortaleza			Recife			Brasília		
ID	Start	End	Total Hosp.	Hosp. COVID-19		Total Hosp.	Hosp. COVID-19		Total Hosp.	Hosp. COVID-19		Total Hosp.	Hosp. COVID-19		Total Hosp.	Hosp. COVID-19		Total Hosp.	Hosp. COVID-19	
				N	%		N	%		N	%		N	%		N	%		N	%
1	24 Feb 2019	09 Mar 2019	15,554	–	–	5897	–	–	2253	–	–	4081	–	–	6118	–	–	3974	–	–
2	10 Mar 2019	23 Mar 2019	17,900	–	–	7160	–	–	2388	–	–	4708	–	–	7382	–	–	4356	–	–
3	24 Mar 2019	06 Apr 2019	17,873	–	–	7198	–	–	2279	–	–	5065	–	–	7603	–	–	4645	–	–
4	07 Apr 2019	20 Apr 2019	17,005	–	–	6582	–	–	2265	–	–	4519	–	–	6786	–	–	4297	–	–
5	21 Apr 2019	04 May 2019	16,938	–	–	6759	–	–	2242	–	–	4873	–	–	7450	–	–	4614	–	–
6	05 May 2019	18 May 2019	18,102	–	–	7034	–	–	2169	–	–	4939	–	–	7860	–	–	4474	–	–
7	19 May 2019	01 Jun 2019	18,130	–	–	7341	–	–	2097	–	–	4938	–	–	8267	–	–	4599	–	–
8	02 Jun 2019	15 Jun 2019	18,124	–	–	7206	,	–	2350	–	–	4862	–	–	7727	–	–	4528	–	–
9	16 Jun 2019	29 Jun 2019	16,919	–	–	6618	–	–	2075	–	–	4496	–	–	7277	–	–	4279	–	–
10	30 Jun 2019	13 Jul 2019	16,831	–	–	7630	–	–	2337	–	–	5184	–	–	8197	–	–	4571	–	–
11	14 Jul 2019	27 Jul 2019	17,975	–	–	6958	–	–	2138	–	–	4851	–	–	7198	–	–	4483	–	–
12	28 Jul 2019	10 Aug 2019	18,157	–	–	7439	–	–	2137	–	–	5080	–	–	7900	–	–	4679	–	–
13	11 Aug 2019	24 Aug 2019	18,455	–	–	7254	–	–	2353	–	–	4721	–	–	8054	–	–	4851	–	–
14	25 Aug 2019	07 Sep 2019	18,221	–	–	7427	–	–	2171	–	–	4978	–	–	8116	–	–	4732	–	–
15	08 Sep 2019	21 Sep 2019	18,970	–	–	7122	–	–	2234	–	–	4867	–	–	7738	–	–	4662	–	–
16	22 Sep 2019	05 Oct 2019	18,466	–	–	7349	–	–	2163	–	–	5060	–	–	8149	–	–	4583	–	–
17	06 Oct 2019	19 Oct 2019	18,438	–	–	7135	–	–	2289	–	–	4770	–	–	7869	–	–	4476	–	–
18	20 Oct 2019	02 Nov 2019	17,802	–	–	7181	–	–	2105	–	–	5053	–	–	7893	–	–	4499	–	–
19	03 Nov 2019	16 Nov 2019	17,422	–	–	7029	–	–	2194	–	–	4470	–	–	7616	–	–	4136	–	–
20	17 Nov 2019	30 Nov 2019	17,670	–	–	6653	–	–	2096	–	–	4741	–	–	7802	–	–	4012	–	–
21	01 Dec 2019	14 Dec 2019	18,842	–	–	7282	–	–	2144	–	–	5125	–	–	7875	–	–	4203	–	–
22	15 Dec 2019	28 Dec 2019	14,473	1	0.0	5327	–	–	1920	–	–	3988	–	–	6335	–	–	3729	–	–
23	29 Dec 2019	11 Jan 2020	14,807	–	–	6181	1	0.0	2132	–	–	4341	–	–	6719	–	–	3800	–	–
24	12 Jan 2020	25 Jan 2020	17,516	1	0.0	6539	1	0.0	2190	–	–	4993	–	–	7697	–	–	4316	–	–
25	26 Jan 2020	08 Feb 2020	18,163	–	–	7260	–	–	2085	–	–	5048	–	–	8004	–	–	4427	–	–
26	09 Feb 2020	22 Feb 2020	18,161	2	0.0	6630	1	0.0	2301	–	–	4927	–	–	7689	–	–	4325	–	–
27	23 Feb 2020	07 Mar 2020	16,409	10	0.1	6020	1	0.0	2018	–	–	4512	1	0.0	6603	4	0.1	4139	2	0.0
28	08 Mar 2020	21 Mar 2020	16,560	76	0.5	5989	11	0.2	2258	4	0.2	4414	3	0.1	6930	19	0.3	4144	2	0.0
29	22 Mar 2020	04 Apr 2020	10,427	729	7.0	4314	183	4.2	1531	189	12.3	3181	70	2.2	4348	85	2.0	3135	18	0.6
30	05 Apr 2020	18 Apr 2020	11,018	1811	16.4	4477	619	13.8	1789	583	32.6	3161	337	10.7	4382	512	11.7	3587	41	1.1
31	19 Apr 2020	02 May 2020	12,451	3432	27.6	4838	1139	23.5	2004	906	45.2	3739	1001	26.8	4779	1151	24.1	3715	106	2.9
32	03 May 2020	16 May 2020	13,444	4159	30.9	4958	1328	26.8	1934	884	45.7	3988	1739	43.6	5302	1692	31.9	4023	264	6.6
33	17 May 2020	30 May 2020	13,762	4017	29.2	5217	1198	23.0	1726	528	30.6	4075	1744	42.8	5433	1610	29.6	3952	397	10.0
34	31 May 2020	13 Jun 2020	14,269	3563	25.0	5314	720	13.5	1804	455	25.2	4044	1063	26.3	5599	1146	20.5	4040	608	15.0
35	14 Jun 2020	27 Jun 2020	13,732	2887	21.0	5084	367	7.2	2066	396	19.2	3945	659	16.7	5681	848	14.9	4171	760	18.2
36	28 Jun 2020	11 Jul 2020	14,099	2730	19.4	5708	304	5.3	2070	335	16.2	4294	414	9.6	6293	818	13.0	3981	916	23.0
37	12 Jul 2020	25 Jul 2020	14,462	2534	17.5	5882	310	5.3	2163	245	11.3	4127	265	6.4	6120	842	13.8	4112	977	23.8
38	26 Jul 2020	08 Aug 2020	14,757	2056	13.9	6268	328	5.2	2076	196	9.4	4276	167	3.9	6488	670	10.3	4288	1049	24.5
39	09 Aug 2020	22 Aug 2020	15,060	1547	10.3	6486	424	6.5	2201	228	10.4	4363	84	1.9	6985	551	7.9	4416	991	22.4
40	23 Aug 2020	05 Sep 2020	15,195	1130	7.4	6559	450	6.9	2174	231	10.6	4252	50	1.2	7065	504	7.1	4082	739	18.1
Before the pandemic (fortnight≤26)	Hospitalizations/ fortnight	Mean (sd)	17,574 (1126)			6930 (522)			2196 (108)			4795 (311)			7589 (558)			4394 (283)		
		Min-Max	14,473 – 18,970			5327 – 7630			1920 – 2388			3988 – 5184			6118 – 8267			3729 – 4851		
		Median	17,937.5			7128.5			2180.5			4870			7732.5			4475		
	Age (years)	Mean (sd)	53.3 (18.1)			55.1 (18.1)			51.1 (18.7)			53.0 (18.8)			53.0 (18.2)			50.8 (18.6)		
		Median	54.0			57.0			51.0			54.0			54.0			50.0		
	Gender (Male)	%	50.8			47.8			53.3			52.4			49.4			50.8		
	Hospital stay (days)	Mean (sd)	5.8 (8.7)			9.0 (12.8)			7.7 (8.7)			8.8 (10.8)			6.5 (7.5)			7.1 (10.2)		
		Median	3.0			4.0			4.0			5.0			4.0			3.0		
	Surgical Hospitalizations	%	46.4			52.0			45.4			52.2			45.9			39.9		
		Min-Max	40.9–48.5			46.1–54.9			39.4–51.4			45.9–55.5			39.2–48.2			35.7–42.5		
	Elective Hospitalizations	%	42.0			44.8			35.7			28.3			37.8			20.8		
		Min-Max	33.1–44.8			35.6–49.2			29.7–42.6			22.8–32.8			28.7–40.7			12.9–24.5		
	ICU Use	%	9.7			6.8			8.0			8.4			8.7			4.9		
		Min-Max	9.3–11.0			6.0–8.5			6.8–9.1			7.1–9.6			7.4–9.7			4.2–5.7		
	Hospital Mortality	%	6.8			10.6			8.8			6.2			6.7			5.4		
		Min-Max	6.1–8.2			9.4–12.7			6.5–11.1			5.5–7.1			5.9–8.1			4.7–6.4		
During the pandemic (fortnight≥27)	Hospitalizations / fortnight	Mean (sd)	13,975 (1760)			5508 (726)			1987 (207)			4027 (416)			5858 (932)			3985 (322)		
		Min-Max	10,427 – 16,560			4314 – 6559			1531 – 2258			3161 – 4512			4348 – 7065			3135 – 4416		
		Median	14,184			5511			2042			4161			5900.5			4061		
	Age (years)	Mean (sd)	54.7 (18.0)			55.8 (18.3)			52.3 (18.7)			53.4 (18.9)			53.8 (18.2)			51.4 (18.5)		
		Median	56.0			58.0			52.0			54.0			55.0			51.0		
	Gender (Male)	%	54.6			51.2			56.1			55.4			51.6			52.1		
	Hospital stay (days)	Mean (sd)	6.7 (8.9)			8.9 (11.1)			8.0 (8.4)			8.7 (9.9)			6.7 (7.2)			6.3 (8.4)		
		Median	4.0			5.0			5.0			5.0			4.0			3.0		
	Surgical Hospitalizations	%	34.2			41.9			32.8			44.8			35.5			34.6		
		Min-Max	25.3–45.6			26.9–52.3			15.5–43.0			28.0–55.7			25.0–46.2			27.6–41.7		
	Elective Hospitalizations	%	26.1			39.1			25.0			18.9			27.2			11.5		
		Min-Max	15.7–40.7			32.0–43.8			14.0–38.1			9.7–25.1			20.1–37.2			7.5–19.7		
	ICU Use	%	15.0			9.6			11.6			11.6			12.2			7.0		
		Min-Max	10.0–17.4			6.3–12.7			7.6–16.1			7.6–15.4			8.7–15.6			5.5–8.6		
	ICU Use *	%	11.7			8.6			8.2			9.3			9.7			5.8		
		Min-Max	9.9–14.1			6.3–10.9			4.3–10.2			7.6–11.3			8.3–11.2			5.0–7.1		
	Hospital Mortality	%	11.4			16.9			16.2			10.9			10.3			7.8		
		Min-Max	7.4–14.9			10.5–26.9			9.6–37.4			5.5–20.8			6.6–15.6			4.9–10.9		
	Hospital Mortality *	%	9.3			14.6			11.8			8.0			8.3			6.5		
		Min-Max	7.3–12.1			10.4–21.6			8.9–26.0			5.4–12.1			6.3–12.1			4.9–9.1		

Source: Ministry of Health - SUS Hospital Information System (SIH/SUS)

* Excluding hospitalizations due to COVID-19

When comparing the indicators before and during the pandemic, the differences in the length of hospital stay in the city of Rio de Janeiro and the use of ICU in Brasília were not statistically significant when COVID-19 hospitalizations were excluded during the pandemic

In the six capitals, comparisons between statistics in the year before (baseline) and in the first six months of the pandemic indicate a reduction in the mean number of hospitalizations per fortnight, increase in the mean age of patients and proportion of males, and, except for Rio de Janeiro, changes (in both directions) in the mean length of stay. The proportion of surgical and elective hospitalizations declined significantly in all cities.

The proportions of ICU hospitalizations spiraled in the pandemic. From baseline to fortnights 27–40, they increased from 9.7 to 15.0% in São Paulo, from 6.8 to 9.6% in Rio de Janeiro, from 8.0 to 11.6% in Manaus, from 8.4 to 11.6% in Fortaleza, from 8.7 to 12.2% in Recife, and from 4.9 to 7.0% in Brasília. It is also interesting to note that, except for Brasília, the increased use of ICU is observed even when COVID-19 hospitalizations during the pandemic are excluded from the comparison.

There were also significant increases in the six capitals with the pandemic regarding hospital mortality, including or excluding COVID-19 hospitalizations from the comparison in the period that includes fortnights 27–40. The high mortality levels observed in Rio de Janeiro and Manaus are noteworthy, both at the baseline and during the pandemic.

**Fig. 1:**
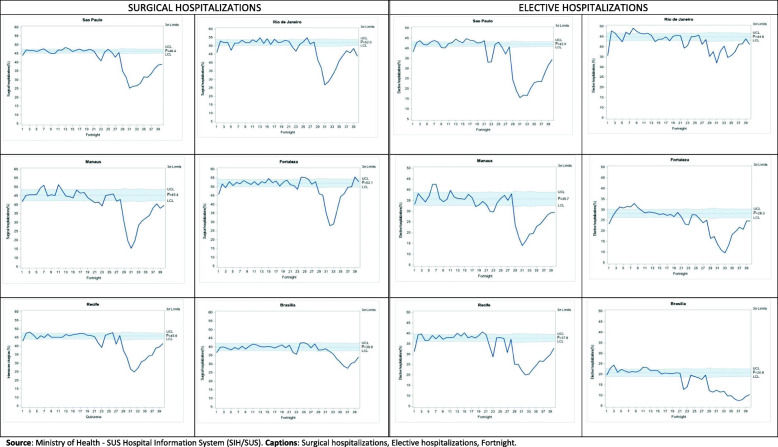
Proportion of surgical and elective hospitalizations to the Unified Health System in six Brazilian capitals, by fortnight, between 24/02/2019 and 05/09/2020. Source: Ministry of Health - SUS Hospital Information System (SIH/SUS). Captions: Surgical hospitalizations, Elective hospitalizations, Fortnight

**Fig. 2:**
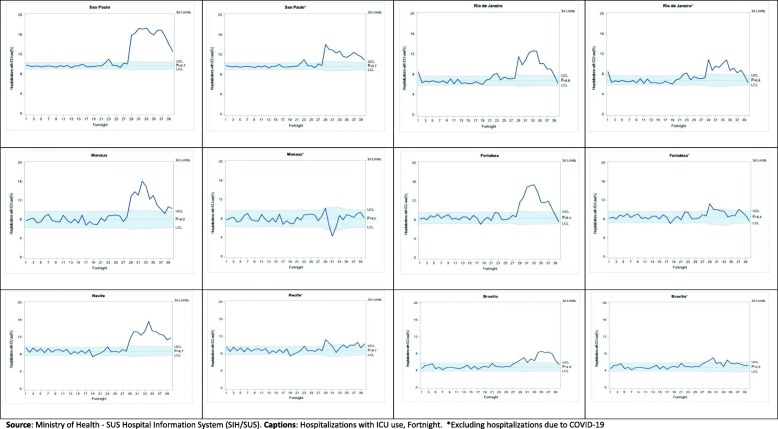
Proportion of hospitalizations with the use of the Intensive Care Unit (ICU) in the Unified Health System in six Brazilian capitals, by fortnight, including and excluding COVID-19 hospitalizations during the pandemic, 24/02/2019 to 05/09/2020. Source: Ministry of Health - SUS Hospital Information System (SIH/SUS). Captions: Hospitalizations with ICU use, Fortnight. *Excluding hospitalizations due to COVID-19

**Fig. 3:**
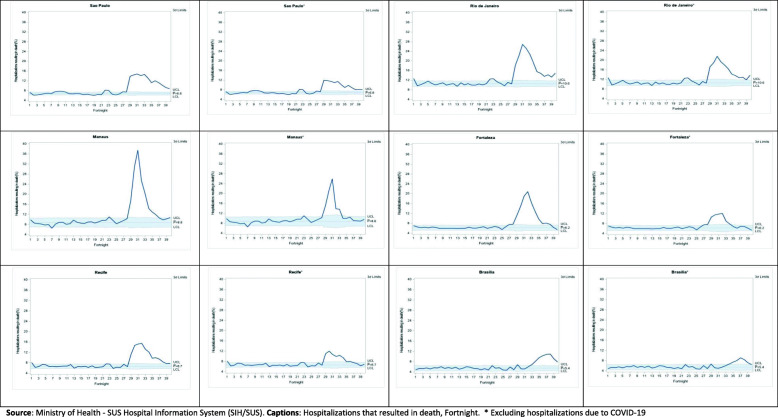
Proportion of hospitalizations that resulted in death in the Unified Health System in six Brazilian capitals, by fortnight, including and excluding COVID-19 hospitalizations during the pandemic, 24/02/2019 to 05/09/2020. Source: Ministry of Health - SUS Hospital Information System (SIH/SUS). Captions: Hospitalizations that resulted in death, Fortnight. * Excluding hospitalizations due to COVID-19

The statistical control charts (Figs. 1, 2, 3) for the proportions of surgical and elective hospitalizations, ICU hospitalizations, and hospitalizations resulting in death clarify the breach of reasonable statistical control observed in the baseline, represented by the year before the onset of the pandemic, from the fortnight in which the pandemic starts in the country. The charts confirm the significant decline in surgical and elective hospitalizations, the increased use of ICUs, and higher hospital mortality. As shown in Table 1, it can also be seen that the increased use of ICU and hospital mortality is observed, albeit to a lesser extent, even when COVID-19 hospitalizations in the pandemic are excluded from the comparison.

The comparison of the number of hospitalizations per fortnight and hospital mortality for ten prevalent diagnostic clusters in the six capitals is shown in Table 2. While selected, it is worth clarifying that diabetes mellitus was among the most frequent diagnoses in hospitalizations only in Manaus, which presents a somewhat differentiated hospitalization profile. Additionally, it is emphasized that six of the ten groups also have high hospital mortality.

**Table 2:** Comparison of the number of hospitalizations, by fortnight and hospital mortality by frequent primary diagnoses in hospitalizations in the Unified Health System in six Brazilian capitals, before and during the COVID-19 pandemic, 24/02/2019 to 05/09/2020

Main diagnosis (ICD-10)	Indicator	Period/Comparison	São Paulo	Rio de Janeiro	Manaus	Fortaleza	Recife	Brasília
Contraceptive (Z30)	Hospitalizations/ fortnight Mean (sd)	Before	462.7 (83.0)	99.4 (13.8)	23.0 (8.6)	31.3 (7.5)	121.6 (21.2)	50.7 (11.6)
		Pandemic	114.6 (117.3)	59.1 (22.8)	15.4 (11.7)	23.4 (6.5)	54.0 (28.5)	29.4 (15.6)
		T-Test (*p*-value)	< 0.0001	< 0.0001	0.0259	0.0017	< 0.0001	< 0.0001
	Mortality (%)	Before	0.0	0.0	0.0	0.1	0.0	0.0
		Pandemic	0.0	0.0	0.0	0.0	0.0	0.0
		Fisher’s Exact Test (p-value)	1.0000	–	–	1.0000	–	–
Septicemia (A40, A41)	Hospitalizations/ fortnight Mean (sd)	Before	408.5 (29.1)	147.8 (22.2)	50.7 (9.1)	70.2 (9.2)	94.0 (16.7)	64.8 (10.9)
		Pandemic	306.1 (55.4)	107.1 (24.4)	37.1 (18.7)	54.6 (20.6)	59.5 (12.2)	55.3 (14.2)
		T-Test (p-value)	< 0.0001	< 0.0001	0.0205	0.0161	< 0.0001	0.0223
	Mortality (%)	Before	59.2	75.7	69.1	62.1	56.3	36.2
		Pandemic	61.7	76.4	71.5	58.0	51.3	26.1
		Fisher’s Exact Test (*p*-value)	0.0045	0.6183	0.3377	0.0521	0.0124	< 0.0001
Cholelithiasis (K80) and Cholecystitis (K81)	Hospitalizations/ fortnight Mean (sd)	Before	538.7 (48.4)	206.8 (30.7)	171.2 (18.2)	151.3 (16.9)	191.9 (24.2)	158.9 (21.9)
		Pandemic	238.1 (121.9)	104.2 (47.1)	95.4 (45.9)	79.5 (46.2)	104.8 (54.6)	117.9 (27.2)
		T-Test (*p*-value)	< 0.0001	< 0.0001	< 0.0001	< 0.0001	< 0.0001	< 0.0001
	Mortality (%)	Before	0.6	1.0	0.9	0.8	1.1	0.4
		Pandemic	1.1	1.8	1.1	1.4	1.6	0.4
		Fisher’s Exact Test (p-value)	0.0046	0.0112	0.6267	0.0477	0.1336	0.8132
Acute myocardial infarction (I21)	Hospitalizations/ fortnight Mean (sd)	Before	429.0 (51.2)	130.1 (19.4)	53.0 (9.0)	82.4 (19.1)	117.4 (21.5)	90.4 (15.1)
		Pandemic	369.2 (58.6)	106.8 (19.2)	40.3 (11.8)	76.0 (24.9)	106.9 (25.2)	89.7 (12.8)
		T-Test (p-value)	0.0018	0.0008	0.0005	0.3715	0.1717	0.8888
	Mortality (%)	Before	8.6	10.1	10.0	12.0	8.9	2.9
		Pandemic	9.5	9.4	12.8	11.8	9.0	3.8
		Fisher’s Exact Test (p-value)	0.0663	0.4992	0.0772	0.8623	1.0000	0.1352
Heart failure (I50)	Hospitalizations/ fortnight Mean (sd)	Before	357.6 (37.6)	95.6 (16.2)	84.0 (13.7)	140.5 (14.9)	183.0 (18.5)	90.3 (15.8)
		Pandemic	273.1 (52.0)	71.9 (24.7)	57.2 (21.5)	85.6 (30.3)	137.4 (44.4)	64.6 (11.1)
		T-Test (p-value)	< 0.0001	0.0008	0.0005	< 0.0001	0.0022	< 0.0001
	Mortality (%)	Before	15.5	20.3	17.9	11.2	9.6	7.8
		Pandemic	18.2	24.3	21.0	14.8	12.1	8.6
		Fisher’s Exact Test (p-value)	0.0002	0.0095	0.0640	0.0011	0.0029	0.4274
Stroke (I60-I64)	Hospitalizations/ fortnight Mean (sd)	Before	434.2 (24.5)	187.0 (20.3)	45.3 (11.5)	170.9 (22.6)	349.2 (24.3)	99.7 (12.5)
		Pandemic	385.8 (36.5)	193.3 (30.9)	40.4 (13.8)	133.3 (24.8)	251.9 (43.3)	97.4 (11.1)
		T-Test (p-value)	< 0.0001	0.4456	0.2305	< 0.0001	< 0.0001	0.5622
	Mortality (%)	Before	15.0	23.1	21.5	12.4	17.6	9.8
		Pandemic	17.7	27.3	21.2	16.9	18.6	11.3
		Fisher’s Exact Test (p-value)	< 0.0001	< 0.0001	0.9503	< 0.0001	0.1619	0.1526
Breast cancer (C50)	Hospitalizations/ fortnight Mean (sd)	Before	295.1 (37.4)	200.1 (17.8)	17.8 (4.1)	69.2 (13.4)	152.5 (22.0)	42.2 (5.4)
		Pandemic	237.9 (39.8)	161.3 (24.4)	15.2 (3.9)	58.5 (11.7)	164.5 (16.8)	41.6 (6.4)
		T-Test (p-value)	< 0.0001	< 0.0001	0.0625	0.0167	0.0821	0.7461
	Mortality (%)	Before	8.0	12.8	11.3	3.0	7.9	9.4
		Pandemic	9.0	14.2	14.6	2.8	4.7	7.7
		Fisher’s Exact Test (p-value)	0.0902	0.1094	0.2563	0.9008	< 0.0001	0.2783
Pneumonia (J12-J18)	Hospitalizations/ fortnight Mean (sd)	Before	550.8 (75.7)	161.0 (23.4)	88.7 (16.2)	134.7 (26.7)	142.7 (18.7)	139.0 (29.2)
		Pandemic	637.9 (149.3)	277.6 (87.3)	68.6 (28.4)	80.8 (44.1)	83.5 (33.0)	136.8 (25.7)
		T-Test (p-value)	0.0570	0.0002	0.0254	0.0005	< 0.0001	0.8163
	Mortality (%)	Before	19.7	29.7	21.0	22.8	13.1	11.8
		Pandemic	19.1	38.1	30.0	28.5	21.1	11.3
		Fisher’s Exact Test (p-value)	0.2834	< 0.0001	< 0.0001	0.0001	< 0.0001	0.5966
Leg (S82), femur (S72), and forearm (S52) fractures	Hospitalizations/ fortnight Mean (sd)	Before	661.3 (35.3)	338.3 (21.3)	92.3 (12.9)	244.8 (26.1)	177.0 (16.0)	145.8 (9.4)
		Pandemic	582.7 (82.4)	335.4 (30.2)	84.4 (26.1)	223.0 (29.8)	160.4 (30.2)	152.3 (17.8)
		T-Test (p-value)	0.0037	0.7244	0.2969	0.0216	0.0724	0.2187
	Mortality (%)	Before	1.5	2.2	1.0	0.6	1.1	0.4
		Pandemic	1.7	3.2	2.0	0.9	1.2	0.3
		Fisher’s Exact Test (p-value)	0.1397	0.0003	0.0275	0.0817	0.7144	0.8252
Diabetes mellitus (E10-E14)	Hospitalizations/ fortnight Mean (sd)	Before	170.1 (12.0)	81.8 (10.1)	73.8 (12.3)	37.0 (7.4)	65.1 (15.0)	64.3 (8.5)
		Pandemic	131.2 (28.8)	69.2 (17.9)	53.4 (18.3)	36.8 (9.5)	45.2 (11.5)	48.1 (8.6)
		T-Test (p-value)	0.0002	0.0267	0.0001	0.9372	0.0001	< 0.0001
	Mortality (%)	Before	4.5	8.1	4.8	5.5	3.4	2.4
		Pandemic	6.0	12.5	9.0	6.4	5.9	1.9
		Fisher’s Exact Test (p-value)	0.0101	0.0002	< 0.0001	0.4859	0.0090	0.5428

**Source**: Ministry of Health - SUS Hospital Information System (SIH/SUS)

ICD-10 = International Statistical Classification of Diseases and Related Health Problems, tenth revision

sd = standard deviation

Table 2 shows a consistent and significant drop during the pandemic in hospitalizations for contraception (ICD-10: Z30), septicemia (ICD-10: A41 and A42), cholelithiasis and cholecystitis (ICD-10: K80 and K81, respectively), heart failure (ICD-10: I50) and, except for Fortaleza, diabetes mellitus (ICD-10: E10-E14) in the six capitals. Acute myocardial infarction hospitalizations decline significantly in São Paulo, Rio de Janeiro, and Manaus and are not statistically different in Fortaleza, Recife, and Brasília. Stroke hospitalizations (ICD-10: I60-I64) and leg, femur, and forearm fractures (ICD-10: S82, S72, and S52, respectively) fell in São Paulo, Fortaleza, and Recife (with borderline statistical significance), not differing from the baseline in Rio de Janeiro, Manaus, and Brasília. In turn, breast cancer hospitalizations (ICD-10: C50) decreased significantly in São Paulo, Rio de Janeiro, and Fortaleza, also showing a drop in borderline statistical significance in Manaus. Recife recorded an increase in borderline significance. Finally, pneumonia hospitalizations (ICD-10: J12-J18) increased in São Paulo and Rio de Janeiro and declined in Manaus, Fortaleza, and Recife.

Hospital mortality in septicemia hospitalizations increased in São Paulo and declined in Fortaleza, Recife, and Brasília. They remained at a very high level, without significant differences, in Rio de Janeiro and Manaus. Cholelithiasis and cholecystitis hospitalization mortality increased in São Paulo, Rio de Janeiro, and Fortaleza. Hospital mortality in hospitalizations due to acute myocardial infarction is not, in general, statistically differentiated in the pandemic, with higher borderline significance recorded in São Paulo and Manaus. Only Brasília showed no increase in hospital mortality in heart failure hospitalizations. Hospital mortality increased significantly in stroke hospitalizations in São Paulo, Rio de Janeiro, and Fortaleza, in pneumonia hospitalizations in Rio de Janeiro, Manaus, Fortaleza, and Recife, and diabetes mellitus hospitalizations in São Paulo, Rio de Janeiro, Manaus, and Recife. A notable reduction in mortality among breast cancer hospitalizations was observed in Recife. Finally, we highlight the significant increase in mortality due to limb fractures in Rio de Janeiro and Manaus, and with borderline significance in Fortaleza.

## Discussion

The study showed significant changes in the patterns of hospital utilization and mortality in the first six months of the COVID-19 pandemic in the six selected capitals. All cities were affected by the pandemic, but a delay in the spike of COVID-19 cases and hospitalizations in Brasilia, compared to the others, probably influenced to some extent the results found.

In general, the reduction in hospitalizations, especially in surgical and elective ones, was expected as the pandemic imposed a reorganization of the existing human and technological resources in hospitals. Further, it required the incorporation of new resources such as the opening of campaign hospitals for the exclusive care of COVID-19 patients.

Blecker et al. [15] identified similar reductions in the U.S. They raised as possible causes for the decrease in the number of hospitalizations the fear of contamination of patients in hospital environments, changes in the behavior of doctors when prescribing hospitalizations, and even lifestyle changes of patients in social distancing.

The reduction observed in the number of hospitalizations for causes such as myocardial infarction, heart failure, and stroke, in some of the cities considered, is in line with what has been observed in other countries such as France, Italy, and the U.S. [26–28]. This reduction may be associated with deaths occurring at homes., without even having had the opportunity to access hospital care, a hypothesis that is supported by a study on the excess deaths during the pandemic in four of the six cities considered here [8]. Also, the heavy COVID-19 demand may have induced losses in the care process due to competition for available hospital resources. It is worth mentioning that in the specific case of the U.S., hospitalizations due to acute events began to recover at the end of the first wave of COVID-19, although those related to chronic diseases generally did not, generating questions about the possible excessive use of hospital care in periods before the pandemic or even better self-care in the context of the pandemic [15].

Concerning fracture-related hospitalizations in São Paulo and Fortaleza, in particular, the decline observed may be partly explained by the decreased occurrence of accidents resulting from the lower circulation of vehicles during periods of more severe restrictive measures or the option to seek care alternatives in the service network specialized in trauma, such as the preference for non-surgical treatments in borderline orthopedic cases for surgical and non-surgical treatment [29]. On the other hand, the number of this type of hospitalization has not changed significantly in Rio de Janeiro and Manaus, with higher mortality. Rio de Janeiro has the highest proportion of the elderly population in the country, registering a high rate of femur fractures in this population, with results that possibly cover most of the mortality observed in the fracture diagnosis group. The vulnerability of the affected population in the context of the pandemic is undeniable. Also, both Rio de Janeiro and Manaus have been scenarios for some of the worst results during the pandemic in the country, and it is plausible, at least partially, to attribute the increased mortality to problems in the quality of services with direct repercussions on patient safety. Although, in general, the analyses focused on comparing the indicators before and during the pandemic in each capital considered and not precisely the comparison between cities, the differences between them are noteworthy, with Rio de Janeiro and Manaus standing out for low mortality indicators at baseline. Manaus is the only city with complex hospital resources in the state of Amazonas and perhaps the Brazilian capital that most dramatically incarnated the health system’s collapse in the face of the pandemic. In turn, while equipped with care structures, Rio de Janeiro has suffered intense scrapping of this equipment, with accumulated severe problems in managing the health system.

Higher levels of pneumonia hospitalizations were observed in São Paulo and Rio de Janeiro, but it is worth questioning whether such elevations could be confused with COVID-19 itself due to the characteristics of the two diseases and testing issues [30]. Concerning cholelithiasis and cholecystitis, an increase in mortality was also detected in hospitalizations in São Paulo, Rio de Janeiro, and Fortaleza. In a scenario of postponement of elective surgeries, this finding may be related to the deteriorated conditions, resulting in urgent cholecystectomies, for example [31]. As in other cases considered here, the possibility that it reflects, to some extent, issues in the health system’s performance and loss of hospital care quality due to the challenges arisen with COVID-19 is not negligible. A significant drop in hospital mortality due to breast cancer was observed only in Recife, which may be indicating a transfer from the place of death, from hospitals to households, mostly requiring further investigations.

We cannot dismiss the hypothesis of excessive use of hospital care due to failing primary care and specialized services before the pandemic. The high volume of hospitalizations related to the diagnostic contraceptive group may be a likely example, and its decline during the pandemic may provide elements for an assessment of the relevance of care at the hospital level. However, predominantly, the pattern of use found in the six capitals favors the hypothesis of the emergence of a currently pent-up demand due to the insufficient use of adequate care, either by the decrease in hospitalizations for specific reasons or the greater use of intensive care, which may reflect greater severity due to postponed care.

Our study has limitations. The approach, descriptive design, and selected indicators allowed us to outline the situation and some changes in hospital care patterns in the pandemic context. However, the disaggregation, standardization, and stratification of the indicators would certainly provide detailed additional information. It was an option to favor this general outlook that is expected to formulate inquiries and outline ways to improve performance. In turn, the data source used, namely, SIH, has gaps such as the specific inclusion of SUS hospitalizations, hindering a more comprehensive analysis considering the population of beneficiaries of health plans using private health services. In the case of large capitals, with concentrations of health plan beneficiaries much higher than the national mean, exclusion can reach levels of up to more than 40.0% of the population, as in the case of São Paulo and Rio de Janeiro. The SIH also does not include cases seen in emergency rooms. Also, to the extent that the study stops at recent hospitalizations, the hypothesis that they may be underreported, even with care taken to mitigate the problem, is not ruled out. Other limitations have to do, on one hand, with the possibility of underreporting COVID-19 cases, given the low testing capacity demonstrated in the country, and, on the other hand, the impossibility of accounting for rehospitalizations, what probably would be relevant in approaching some of the diagnoses selected.

Despite the limitations mentioned, especially in a context such as the pandemic, which imposes significant challenges for the health system, it is worth emphasizing the importance of having a national database, which, in global terms, covers about 75% of the Brazilian population, and is made available relatively quickly. This work joins others already published in other countries and contributes by examining in detail effects of the COVID-19 pandemic on hospitalizations and hospital mortality in the six selected Brazilian capitals, considering the universe of hospitalizations due to COVID-19 and other conditions covered by the SUS.

## Data Availability

The manuscript was based on data publicly available at https://datasus.saude.gov.br/transferencia-de-artigos/, obtained on January 19th 2021. The dataset analyzed and the SAS® programs employed may be requested to the corresponding author.
